# Intervention Effects of Physical Activity on Type 2 Diabetic Patients Potentially Infected with COVID-19

**DOI:** 10.3390/medicina59101772

**Published:** 2023-10-05

**Authors:** Lihua Yu, Sainyu Guo, Wen Ji, Hailian Sun, Seongno Lee, Deju Zhang

**Affiliations:** 1 College of Arts and Sports, Hanyang University, 222 Wangsimni-ro, Seongdong-gu, Seoul 04763, Republic of Korea; yulihua0210@163.com (L.Y.);; 2 Institute of Public Foundations, University of Health and Rehabilitation Sciences, Qingdao 266000, China; 3 College of Arts and Sports, Myongji University, Seoul 04763, Republic of Korea; 4 Food and Nutritional Sciences, School of Biological Sciences, The University of Hong Kong, Pokfulam Road, 0000, Hong Kong

**Keywords:** COVID-19, physical activity, diabetes, immunity, SARS-CoV-2

## Abstract

The coronavirus disease 2019 (COVID-19) pandemic has clearly had a great influence on the lifestyles of the population, especially on patients with type 2 diabetes mellitus. During the COVID-19 outbreak, many countries/regions implemented social-isolation measures, leading to an increase in negative behaviors and impairing the capability of diabetic patients to resist COVID-19, ultimately causing severe prognoses. Moreover, as the epidemic progressed, multiple studies emphasized the significance of physical exercise in the management of type 2 diabetic patients infected with COVID-19. In this study, we selected research from 1 December 2019 to 9 August 2023 that focused on COVID-19-infected diabetic patients to investigate the impact of type 2 diabetes on the immune functions, inflammation factor levels, lung injuries, and mental disorders of such patients, as well as to assess the risk of novel coronavirus pneumonia in these patients. Additionally, the effects of high-intensity, moderate-intensity, and low-intensity exercises on novel coronavirus pneumonia infection in type 2 diabetic patients and the mechanisms of the effects of such exercise were considered. We concluded that elderly diabetic patients with COVID-19 should perform low-intensity exercises to facilitate their recoveries. This study offers guidance for a proper understanding of the dangers of diabetes and the use of appropriate measures to reduce the risk of novel coronavirus pneumonia infections in type 2 diabetic patients.

## 1. Introduction

Coronavirus disease 2019 (COVID-19) is a type of coronavirus (COV). It was first identified in the 1960s and caused large-scale epidemics in 2002–2003 (severe acute respiratory syndrome) and 2012 (Middle East respiratory syndrome) [1]. COVID-19 was declared a pandemic by the World Health Organization (WHO) in January 2020 [2]. By 9 November 2022, there were over 630 million confirmed cases and over 6.58 million deaths caused by COVID-19. 

Severe acute respiratory syndrome coronavirus 2 (SARS-CoV-2) was the causative agent of the COVID-19 pandemic. SARS-CoV-2 is an enveloped virus with a great, single-stranded, positive-sense RNA genome; it consists of a spike protein (S), a membrane protein (M), a nucleocapsid protein (N), and an envelope protein (E) of the genus coronaviruses [3], which can be transmitted from person to person in the state of a respiratory droplet and aerosol [4]. Conventionally, the main modes of transmission are coughing, sneezing, droplet inhalation, and touching mucous membranes [5]. Novel coronavirus pneumonia is a very harmful pathogen that can induce respiratory failure, pneumonia, multi-organ failure, and even death [6]. 

Some diseases, such as hypertension, diabetes, and cardiovascular disease—and especially, type 2 diabetes—have been continuously demonstrated as risk factors for mortality and poor prognosis in COVID-19 patients [7]. Diabetes mellitus not only contributes to high mortality and poor prognosis in patients with novel coronavirus pneumonia, but also has deleterious effects on the host immune systems, lungs, moods, and inflammation levels of patients [8,9]. In several studies comparing non-diabetic patients infected with COVID-19 to diabetic patients with COVID-19, non-diabetic patients infected with COVID-19 exhibited higher survival rates [10]. Therefore, understanding the mechanisms of damage and the negative effects of COVID-19 on type 2 diabetes patients is of crucial importance for the prophylaxis of novel coronavirus pneumonia infection in the diabetic population.

Despite the development of various vaccines for the treatment of COVID-19, there will always be a need for more cost-effective ways to prevent and treat the SARS-CoV-2 virus and the ensuing COVID-19 disease, especially in poor countries [11]. Physical exercises, including high-intensity, moderate-intensity, and low-intensity exercises, are important tools for the prophylaxis and treatment of diabetes and novel coronavirus pneumonia infection [12]. Such exercises are valuable, safe, and effective for the management of diabetic patients, and could be extensively used during the COVID-19 outbreak to prevent patients from being infected. The beneficial effects of exercise are reflected in improved immune function [13], the suppression of lung damage, and reductions in inflammation factors [14,15], anxiety, and depression [16]. In diabetic patients, exercise has been demonstrated to play a role in controlling blood glucose levels and boosting immunity [17]. As suggested by Laddu, Lavie [18], moderate-intensity physical activity can effectively protect individuals against viral respiratory infections. Sellami, Gasmi [19] also indicated that high-intensity exercise can promote immunity and prevent diseases. Endurance training, including treadmill walking, cycling, and swimming, is a kind of exercise that can improve the aerobic system and cardio-respiratory function, as well as the exercised muscles. Resistance training, also known as strength training, consists primarily of various forms of exercise, with or without equipment, that force skeletal muscles to contract in response to some types of force that resist movement, including free weights, weight machines, and elastic resistance bands [17]. In this study, non-resistance exercises with multiple benefits were included, considering both the intensity of exercise required and the needs of diabetic patients infected with COVID-19, to improve cardiorespiratory fitness, enhance muscular endurance, improve blood circulation, and reduce the risks of obesity and diabetes.

We considered the link between exercise intensity and COVID-19 infection in type 2 diabetic patients. How does each type of exercise affect the health conditions of type 2 diabetes patients? We investigated the significance of different intensities of non-resistance exercises in type 2 diabetes in enhancing the immune function and in protecting against possible infections such as COVID-19. We also explored the potential mechanisms of these training methods, as possible interventions that could be used in the current stage of the COVID-19 pandemic. Our findings will be of interest in choosing the optimal exercise intensities for patients with type 2 diabetes and COVID-19 infections.

## 2. The Relationship between COVID-19 Infection and Diabetes

Diabetes, a disease of the endocrine system that is characterized by abnormally high blood glucose levels, is now one of the most common and fastest-growing diseases worldwide [20]. According to Lukmanto, Suharjito [21], the number of patients with diabetes reached 415 million in 2015, and this number is expected to increase to 642 million in 2040. Diabetes can be divided into four types: type 1, type 2, gestational diabetes mellitus, and other types of diabetes [22,23]. Numerous studies have revealed that diabetic patients are positively correlated with a higher susceptibility to certain infectious diseases, including H1N1 influenza, staphylococcus aureus, and mycobacterium tuberculosis, which may be attributed to a disordered immune system [24,25]. In addition, COVID-19 has been thought to result in diabetes, further worsening the control of glycemia in patients with pre-existing diabetes [26]. Therefore, the global epidemic of COVID-19 might have direct implications for the progression, treatment, and prognosis of type 2 diabetes mellitus. 

In this context, we considered some studies of the relationship between diabetes and COVID-19 infection and the proportion and prognosis of diabetic patients among those infected with COVID-19, with the aims of improving the awareness of high infection and mortality rates caused by COVID-19 in type 2 diabetic patients and, ultimately, strengthening the protection of frail patients.

Many factors may contribute to the progression and poor prognosis of COVID-19, such as smoking, obesity, cardiovascular diseases, and diabetes [27]. Several researchers reported that diabetes had deleterious effects on host immunity and was highly associated with increased susceptibility to pulmonary infection [28,29], suggesting that hyperglycemia may be a major contributor to the increased risk of novel coronavirus pneumonia infections and to the enhanced odds of death in patients with novel coronavirus pneumonia. Emerging studies indicate that COVID-19 infection is more common in diabetic patients, although the prevalence rate varies in different studies and in data from different countries. The database from the Chinese Centers for Disease Control and Prevention (CDC) suggested that 5% of COVID-19 patients were also diagnosed with diabetes, while this value was 13% and 4% for hypertension and cardiovascular disease, respectively [30]. Other studies from China indicated that diabetes was one of the most common comorbidities, with co-occurring cases of diabetes and COVID-19 accounting for 22%, 16.2%, and 12% of all cases in the studies of Yang, Yu [31], Guan, Ni [32], and Zhang, Dong [33], respectively. A study conducted by Italian scholars revealed that diabetic patients accounted for nearly 17% of the 1043 COVID-19 patients that were studied [34]. Indian researchers found, similarly, that the prevalence of hypertension, diabetes, and cardiovascular disease were 21%, 11%, and 7% in COVID-19 cases, respectively [35]. A US study reported the highest correlation between COVID-19 and diabetes among many comorbidities, with diabetics accounting for 58% of the COVID-19 patients investigated [36]. In contrast, a Chinese study reported the lowest correlation between COVID-19 and diabetes among several comorbidities, with diabetics accounting for 5.3% of the COVID-19 patients investigated [30]. Studies from different countries and different teams have demonstrated that diabetic patients are more likely to be infected with COVID-19, and the variation in the percentage of diabetics in patients with COVID-19 might be attributed to individual differences. Furthermore, a nationwide study conducted in England reported that there were independent correlations between type 2 diabetes and the higher odds of in-hospital death with the novel coronavirus pneumonia [37].

In addition to being highly associated with the prevalence of diabetes, COVID-19 is also positively correlated with poor prognoses of diabetes. The serious adverse prognoses involve severe diabetic complications, ICU admissions, and high mortality rates [38,39], as well as long-term risks such as fatigue, dyspnea, muscle and joint pain, and inability to concentrate [26]. In addition, studies have shown a higher risk of new-onset hyperglycemia in patients with COVID-19 infection, which in turn leads to worsening glycemic control in patients with pre-existing diabetes [26]. COVID-19 infections are commonly found in the diabetic population, leading to poor prognosis in these patients, including diabetic complications, ICU admissions, and new-onset diabetes. New-onset diabetes further causes elevated blood glucose, while diabetic complications may lead to a weaker immune system, thereby reducing the resistance of patients to the novel coronavirus pneumonia and further increasing the risk of novel coronavirus pneumonia infection.

One review revealed that the overall symptoms of patients suffering from both diabetes and novel coronavirus pneumonia were not significantly different from those of patients with COVID-19 infection only [40]. However, 14.5% of the subjects were diabetic patients who exhibited acute respiratory distress syndrome with severe symptoms, and who had a higher death rate than other COVID-19 patients. Similarly, a retrospective study conducted by Roncon, Zuin [39] showed that diabetes mellitus was the second-most-frequent comorbidity among 1382 patients. That study also reported that COVID-19 patients with diabetes had a higher risk of ICU admission and mortality risk, with odds ratios of 2.79 and 3.21, respectively. Analysis of individuals infected with novel coronavirus pneumonia demonstrated that diabetic patients were associated with an increased possibility of novel coronavirus pneumonia infection and death, as well as with a poorer prognosis. 

Although most studies support a correlation between diabetes and novel coronavirus pneumonia infection, there are some scholars who believe that diabetes may not increase the risk of COVID-19 infection [26]. This can be attributed to the fact that diabetics are more aware of the risk of adverse outcomes of infection and more readily follow non-pharmacological measures, such as wearing masks and keeping social distance. Therefore, a more in-depth study of the relationship between type 2 diabetes and novel coronavirus pneumonia is needed to offer guidance on the use of medications, as well as to reduce the possibility of COVID-19 infection in type 2 diabetes patients.

## 3. Reasons for the Increased Diabetic Susceptibility to COVID-19 Infection

**Figure 1:**
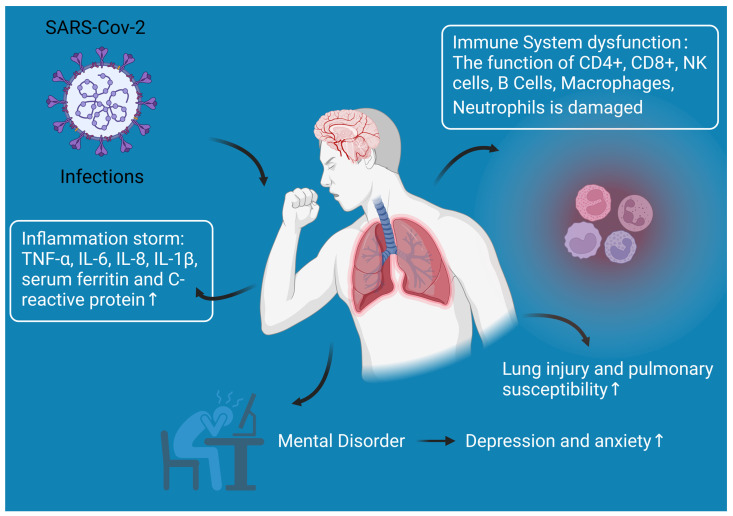
Reasons for the increased susceptibility of diabetic pateints to COVID-19 infection. After entry into the human body, the SARS-CoV-2 virus causes damage to the function of CD4+ T cells, CD8+ T cells, NK cells, B cells, macrophages, and neutrophils of the immune system and leads to an inflammatory storm (accumulation of TNF-α, IL-6, IL-8, IL-1β, serum ferritin, and C-reactive protein) in the human body. In addition, diabetic patients infected with COVID-19 are susceptible to lung injury and to mental disorders such as anxiety or depression.

A variety of changes occur in diabetic patients that make them more susceptible to COVID-19 infection, as shown in Figure 1.

### 3.1. Immune System Dysfunction

Generally, the human body can protect itself against attacks by toxins, parasites, and microorganisms (such as bacteria, viruses, and fungi) [41]. In normal conditions, the immune system of the human body could prevent pathogens from penetration, but it may not function properly in patients with some conditions, such as obesity, diabetes mellitus [42], hypertension, cardiovascular disease [43], and depression [44], resulting in the entry of bacteria, viruses, and fungi into the body and, finally, leading to the development of a series of diseases [41]. Specially, diabetes exerts negative effects on the immune system, including disordered inflammation, impaired innate immunity, and declining adaptive immunity [45]. According to the study of Frydrych, Bian [42], patients with type 2 diabetes are highly associated with being physiologically frail and comorbidly challenging, and they are also recognized as the largest population that suffers from post-infection complications and increasing long-term mortality. Additionally, several scholars have reported that diabetes is highly correlated with an increased risk of viral infections, urinary tract infections, soft-tissue infections [46,47], skin infections [45], and secondary infections [48] that may result in sepsis, renal failure, and death. The respiratory infections caused by viruses in patients with diabetes raise increasing concerns in light of the spread of novel coronavirus pneumonia. Fang, Karakiulakis [49] and Navand, Soltani [50] stated that the rate of influenza infection in patients with diabetes was six times higher than the rate in the non-diabetic population, and diabetes was highly correlated with an increased risk of viral respiratory diseases such as coronavirus (the prevalence of diabetes among COVID-19 patients was around 9.7%). These studies have indicated that infection is a common problem in individuals with diabetes, as a result of the inability of their defense systems to fight off invading pathogens. Physical changes induced by diabetes that may affect COVID-19 infection are shown in Table 1.

There is an altered proliferation of T cells and macrophages and an impaired function of NK cells and B cells in patients with diabetes, suggesting innate and adaptive immune abnormalities [61]. In addition, the underlying innate and adaptive immune system disorders in patients with diabetes promote infectious complications, impair sepsis recovery, and increase long-term mortality [62]. Diabetes is a complex clinical syndrome in which patients exhibit persistent hyperglycemia in the presence of reduced insulin secretion and sensitivity, causing aberrant metabolic changes, including an increased accumulation of advanced glycosylation end products and flux through the polyol and hexosaine (2.4-fold) pathways, as well as activation of protein kinase C isoforms [42,63]. Finally, an approximately 1.7-fold upregulation of the NRLP3 pathway was observed in individuals with diabetes mellitus, which may increase their susceptibility to COVID-19 infection [64,65]. These abnormal metabolic changes cause an increase in the production of superoxide, thereby activating inflammatory pathways, disrupting immune systems, damaging lung tissues, worsening diabetes-related comorbidities, and, ultimately, leading to increased infectivity and virulence of SARS-CoV-2 in diabetes patients [4].

#### 3.1.1. Abnormal T Cell Homeostasis

T cells can be classified as helper (CD4+) T cells and cytotoxic (killer, CD8+) T cells. They play an essential part in the development of diabetes mellitus [66,67]. Patients with type 2 diabetes mellitus generally exhibit over-activated and/or abnormally differentiated T cells and the activation of the inflammatory pathways [55,57], indicating that the normal immune system is disrupted by high blood glucose content and causing the organism to be more susceptible to external viral infections. Helper (CD4+) T cells can be further subdivided into pro-inflammatory Th1 and Th17 and anti-inflammatory Th2 and Foxp3+ regulatory T cell subsets, according to different functions and the types of cytokines produced [62]. Studies have shown that in the peripheral blood and the adipose tissue of type 2 diabetes mellitus, helper (CD4+) T cells are more easily polarized to pro-inflammatory Th1 cells and Th17 cells, while the polarization of anti-inflammatory Th2 cells is significantly suppressed [68,69]. The imbalance in the differentiation of helper (CD4+) T cells is also highly associated with an imbalance distribution of inflammatory factors. The study of Zhou, Chi [8] reported that in patients infected with SARS-CoV-2, the quantity of total T cells and CD4+ and CD8+ T cell subsets were largely suppressed and functionally exhausted, while the control and clearance of pathogens were weakened in patients with diabetes mellitus, causing the inhibition of immune defenses in response to the invasion of COVID-19. Asadikaram, Ram [70] and Sireesh, Dhamodharan [71] reported that Treg cells could produce CD4, CD25, and the forkhead family transcription factor Foxp3, which only account for 5–20% of the CD4+ compartment and play a crucial role in the suppression of effector T cell responses and in the limitation of inflammation [72]. As suggested by Li, Zheng [73], patients with type 2 diabetes mellitus exhibited lower levels of Treg/Th17 (0.47) and Treg/Th1 (0.04) than normal individuals. Therefore, maintaining the balance between Treg, Th1, and Th17 cells is extremely important for the immune response in diabetic patients, which contributes to the prevention of novel coronavirus pneumonia infection in such patients.

CD8+ T cells also play an indispensable role in the process of adaptive immunity against infections via synthesizing pro-inflammatory cytokine IL-17 and producing cytokines, including TNF-α and IFN-γ [51,74]. Several scholars reported that decreased levels of CD8+ T cells (nondiabetic: 328 n/μL, type 2 diabetes: 203 n/μL) and increased proportions of inflammatory cytokines (the levels of IL-4, IL-6, IL-10, TNF-α, and IFN-γ in nondiabetic were 3.64, 5.27, 4.50, 3.83, 3.59 pg/mL, respectively, while the levels of IL-4, IL-6, IL-10, TNF-α, and IFN-γ in type 2 diabetes were 3.67, 12.10, 5.05, 6.43, and 8.83 pg/mL, respectively) were measured in patients with type 2 diabetes mellitus [51,55]. These pro-inflammatory cytokines were distributed in numerous inflammatory tissues, which could aggravate the inflammatory response and cause immune system imbalance. As suggested in a recent study, glucose metabolism disorder highly impaired the antiviral capacity of CD8+ T cells (the proportion of endogenous virus-specific cells decreased from 5% in normal mice to 4% in diabetic mice) [75]. Specifically, type 2 diabetes damages CD8+ T cells mainly by affecting cell metabolism, memory formation, cytokine production, or recall responses. Therefore, type 2 diabetic patients are more likely to be infected by novel coronavirus pneumonia due to the impaired antiviral capacity of CD8+ T cells.

#### 3.1.2. Dysregulation of Natural Killer (NK) Cells

NK cells can be classified into two main subtypes. They have been shown to play an essential role in anti-tumor regulation, hemopoietic regulation, and the primary control of acute viral infections; their dysfunction is thought to be highly associated with an increased risk of multiple infections [76,77]. The activity of NK cells could be regulated by activating receptors, such as NKp30, NKp44, NKp46, NKG2C, and NKG2D. These receptors can attach to the ligands that are present on the surface of infected cells, finally causing the removal of invading bacteria [78]. According to previous studies, persistent hyperglycemia directly affected NK cell defects, thereby damaging their function in preventing acute viral infection in the body [79]. Therefore, it can be speculated that the dysregulation of T cells and NK cells induced by diabetes, along with bacterial coinfection, are crucial causes of the exacerbation of the SARS-CoV-2-infected health condition and the death of patients. A study by Han, Ma [80] found that, compared to non-diabetic COVID-19 patients, the counts of total T lymphocytes (non-diabetic patients: 962.0 × 10^6^/L, diabetic patients: 448.0 × 10^6^/L), CD4+ T cells (non-diabetic patients: 583.0 × 10^6^/L, diabetic patients: 204.0 × 10^6^/L), CD8+ T cells (non-diabetic patients: 352.0 × 10^6^/L, diabetic patients: 115.0 × 10^6^/L), and NK cells (non-diabetic patients: 252.0 × 10^6^/L, diabetic patients: 35.0 × 10^6^/L) were significantly suppressed in diabetic patients with novel coronavirus pneumonia. This finding suggests that the immune system can be highly affected by the disruption of glucose metabolism in diabetic patients, which, in turn, reduces the body’s ability to defend itself against novel coronavirus pneumonia.

#### 3.1.3. Impaired B-Cell Function

B cells exert essential responsibilities in innate and adaptive immunity. They can act as antigen-presenting cells and produce cytokines in response to stimuli [81]. In addition, B cells are known to play a central role in the development of diabetes via the production of IgG antibodies, as well as in the activation of T cells and macrophages [82]. Initial infection by a pathogen results in the proliferation of antigen-specific T and B lymphocytes to control or kill the pathogen [83]. Finally, the immune system will develop a memory pool of antigen-specific adaptive immune cells, creating long-term protection against secondary encounters [84]. Thus, when identical or antigenically similar pathogens attack the human body a second time, the rapid memory of the humoral and cellular arms of the adaptive immune system will be activated [84]. Several researches have also shown that reinfection with the same influenza virus strain can be inhibited or even prevented by antibody-mediated neutralization of viral particles (sterilizing immunity) in immunologically active hosts [85].

However, numerous studies have shown that type 2 diabetes impairs B-cell function and the accumulation of antibodies, mainly in the form of impaired responsiveness toward viral infection, an enhanced production of pro-inflammatory IL-8, and a failure to secrete anti-inflammatory IL-10, all of which are highly associated with inflammatory disease resolution [86,87]. Moreover, Wu, Huang [87] reported that the effective clearance of novel coronavirus pneumonia needs both the action of CD8+ effector T cell-mediated effective killing of virally infected cells and CD4+ T cell-dependent enhancement of CD8+ T and B cell responses. As a result, impaired B-cell function affects the response of the immune system to the COVID-19 strains, impeding the interaction between B-cells and T-cells, ultimately leading to the invasion of COVID-19 into the human body and causing positive infection and delaying the clearance of the COVID-19 virus in diabetic patients, eventually causing long-term viral infections and ongoing symptoms [84,88]. In addition, B-cell dysfunction caused by type 2 diabetes is highly associated with secondary encounters of the virus; therefore, diabetic patients are expected to be more likely to have a secondary infection with SARS-CoV-2 [42]. However, there is insufficient research regarding the link between secondary COVID-19 infection and B cell dysfunctions in type 2 diabetic patients, and further studies are required to verify the results.

#### 3.1.4. Inhibition of Macrophage Phagocytosis

Macrophages are key mediators of inflammation. They are characterized by multiple surface markers and cytokine secretory patterns [62]. Generally, macrophages can be divided into two types, M1 cells or M2 cells, according to their surface phenotype [89]. The former type is triggered via several pro-inflammatory mediators, including LPS or IFN-γ, IL-6, IL-1β, and TNF-α, while the latter type is triggered by IL-4 and IL-13, thus secreting anti-inflammatory IL-10 and IL-1 and, then, blocking IL-1β and iNOS, which are important for tissue repair, angiogenesis, and the resolution of inflammation [90].

Diabetes mellitus can influence the function of macrophages, thereby decreasing their capacity for bacteria recognition and phagocytosis [91,92]. As indicated in the study by Restrepo, Twahirwa [91], chronic hyperglycemia was greatly linked to the impairment of phagocytosis, which can be reflected by drawbacks in complement and Fcγ receptors on isolated monocytes (complement-mediated and FcγR-dependent phagocytosis were inversely associated with chronic hyperglycemia, with β-coefficients being −3.17 and −3.32, respectively, *p* < 0.05). In addition, the increased susceptibility of patients with diabetes to pathogens may be attributed to the activation of effector mechanisms for bacterial killing, which includes the expression of pro-inflammatory cytokines, reactive oxygen species (ROS), and lysosomal enzymes. Pavlou, Lindsay [92] revealed that the antibacterial activity and phagocytosis of macrophages obtained from both mice bone and in their abdominal cavity were greatly suppressed in mice with diabetes. Specifically, the phagocytosis of bone marrow-derived macrophages and peritoneal macrophages were reduced by approximately 43% and 35%, respectively, after 2 h of exposure to a hyperglycemic environment, compared with the untreated group. In contrast, the bactericidal function of bone marrow-derived macrophages decreased by 5.38-fold after 2 h of exposure to hyperglycemic environment. These studies demonstrated that the diminished viral resistance to COVID-19 in diabetic patients may be attributed to the fact that diabetes impaired the function of macrophage.

#### 3.1.5. Dysfunction of Neutrophils

The presence of neutrophils is of vital importance for the containment and eradication of viruses. They mainly exist in the bone marrow and exhibit antibacterial activity due to their specific proteins, including myeloperoxidase and calprotectin. According to the study of Frydrych, Bian [42], patients with type 2 diabetes exhibit defects in almost all functions. Neutrophils migrate to the inflammation sites, release cytosolic proteases, exhibit phagocytosis, and generate large amounts of ROS, thereby promoting apoptosis. Furthermore, Baggiolini, Dewald [93] observed enhanced TNF-α, IL-1β, and IL-8 levels in neutrophils in patients with diabetes. The invasion of COVID-19 could impair the immune system of individuals with diabetes, especially the neutrophils, which can be reflected by their multiple function defects, such as migration to sites of inflammation, accumulation of ROS and apoptosis. These observations demonstrated that patients with diabetes had a higher possibility of neutrophil dysfunction, thereby upregulating the risk of infections.

### 3.2. Lung Injury and Pulmonary Susceptibility

The novel coronavirus pneumonia attacks human lungs, causing abnormalities in the physiology and structure of lung tissue as well as impairing the function of the lungs [94]. Meanwhile, several studies have also shown that diabetes is highly correlated with these abnormalities, suggesting that diabetes not only induces immunodeficiency, but also facilitates lung damage [8]. Zhao, Shi [95] demonstrated that type 2 diabetes mellitus was positively related to a high risk of pathogen infection, which caused inflammatory infiltration, cell apoptosis, and fibrosis of lung tissues of mice, ultimately causing lung injury and increasing pulmonary susceptibility. Kruglikov, Shah [96] also stated that the invasion of the virus facilitated the interactions between ACE2 deficiency and diabetes, thus suppressing the synergistic effect between endothelial and gut barrier function, thereby inducing greater lung damage and pulmonary susceptibility in patients infected with COVID-19. Moreover, as suggested by Zheng, Wu [97] and Al-kuraishy, Al-Gareeb [98], diabetes also induced the accumulation of ROS and pro-inflammation factors (oxygen saturation, serum ferritin, and C-reactive protein were 94.62%, 475.92 ng/mL, and 48.54 mg/L, respectively, in diabetic patients with COVID-19, whereas the values for these parameters were 98.99%, 90.51 ng/mL, and 3.16 mg/L, respectively, in healthy subjects with COVID-19), leading to lung injuries in individuals with diabetes, including an impaired respiratory system and interstitial lung injury. However, how SARS-CoV-2 further exacerbates lung injury and pulmonary susceptibility caused by diabetes needs to be further investigated.

### 3.3. Inflammation Storm

A large number of studies showed that diabetic patients suffered from long term low-grade chronic inflammation, which can trigger cytokine storms that may result in severe consequences of novel coronavirus pneumonia and, ultimately, death [8]. Several scholars demonstrated that diabetic patients infected with COVID-19 are associated with severe inflammation and higher mortality. In addition, the inflammation markers, including serum ferritin and C-reactive protein, largely increased in diabetes patients infected with SARS-CoV-2, which could be deduced from the fact that diabetic patients infected with COVID-19 had greater levels of serum ferritin (1255.0 vs. 372.8 µg/L), C-reactive protein (57.9 vs. 17.3 mg/L), IL-2 (808.0 vs. 565.0 U/mL), IL-6 (29.6 vs. 12.7 pg/mL), IL-8 (21.0 vs. 12.1 pg/mL), IL-10 (21.0 vs. 5.0 pg/mL), and TNF-α (11.0 vs. 8.4 pg/mL) than non-diabetic patients infected with COVID-19 [99]. Fedullo, Schiattarella [100] found that diabetes patients infected with COVID-19 had higher concentrations of IL-6. Targher, Mantovani [101] reported that the concentrations of C-reactive protein increased to 14.1 mg/L in diabetic patients infected with COVID-19, which suggested the activation of the monocyte–macrophage system, which is a central part of the cytokine storms. Thus, both diabetes and novel coronavirus pneumonia may synergistically promote the inflammatory response in the human body. Moreover, individuals with diabetes tend to have more comorbidities as a result of injured target organs. If diabetic patients then suffer from the invasion of novel coronavirus pneumonia, it may cause more severe inflammation, a hypercoagulable state, and even hypoxia, ultimately resulting in a rapid progression of novel coronavirus pneumonia and higher mortality [99].

### 3.4. Other Responses

Individuals with diabetes often suffer from two common mental health conditions: depression and anxiety [102]. Both conditions increase the risk of death, poor management of diabetes, and diabetes-related complications, thereby worsening the disease progress. Prolonged depression and anxiety lead to a weaker immune response, which, in turn, increases the risk of novel-coronavirus-pneumonia infection [103,104]. Furthermore, studies have also shown that the prevalence of novel coronavirus pneumonia increases the risk of depression (27.0%, 41.4%, and 63.1% for low-, medium-, and high-level stressors, respectively) and anxiety (19.3%, 34.6%, 52.2%, respectively), which further reduces the immunity of patients [105]. Additionally, diabetes decreased the counts of the dendritic cells by 53% and inhibited the proportion of γ-delta T cells [106,107], which, in turn, affected the innate immunity and adaptive immunity of individuals, ultimately causing more severe novel-coronavirus-pneumonia infection. Nevertheless, there is no experimental data to confirm this view.

## 4. Physical Activity Improves the Resistance of Diabetics to COVID-19 Infection

Exercise has been reported to exhibit well-established benefits for both healthy individuals and those with various diseases [108]. In particular, individuals with type 2 diabetes have been shown to benefit from exercise, as it can improve their immune function [109], lung function [110], the level of anti-inflammatory cytokines such as IL-4, IL-10 and lipocalin, and health-related quality of life [111,112], while reducing levels of inflammatory factors (TNF-α, IL-1β, and IL-6) and the emotional burden [111,113], thus maintaining a relatively good physical condition and a controlled glycemic status and, ultimately, protecting against COVID-19 virus invasion. In diabetic patients infected with COVID-19, exercise may prevent the worsening of immune dysregulation and lung damage by restoring the proliferation and viability of T cells, macrophages, NK cells, and B cells and reducing the accumulation of ROS, TNF-α, serum ferritin, and C-reactive protein [80,99], thus improving patient outcomes and speeding recovery. Additionally, diabetic patients participating in exercises have been shown to have acute improvements in insulin sensitivity and decreased body weight and blood glucose, blood lipids, and blood pressure levels [114,115]. As indicated in Section 3, the improved hyperglycemic and health status of diabetic patients contribute to restoring the function of the immune system, reducing lung injury and lung susceptibility, and lowering inflammatory factor levels, which eventually prevent the infection of COVID-19 and relieve the symptoms of patients after infection.

However, the emerging pandemic of novel coronavirus pneumonia induced by the SARS-CoV-2 limits people’s physical activity participation, which poses a major health, and economic burden worldwide. In many countries, many restrictive measures, including quarantine, the suspension of social activities, and the closure of public areas, have been taken to prevent the spread of the COVID-19 virus. However, these restrictive measures decrease or even eliminate public access to physical activities, resulting in a decrease in populations’ levels of physical activity during the COVID-19 pandemic [116]. The study of Amini, Isanejad [117] showed that the physical activity of the Iranian population decreased significantly and that about 78% of the subjects could not meet the guidelines of physical activity during the COVID-19 pandemic. Similar observations were reported by Guthold, Stevens [118], who found that approximately 33% of the adult population of Iran did not meet the standard guidelines of physical activity during the period of the COVID-19 pandemic. In addition to lack of exercise, many scholars reported that a large number of populations exhibited higher prevalences of anxiety and depression during the COVID-19 pandemic, which may have arisen from the fear of contracting the virus, the lack of effective treatment, the risk of death caused by the virus, and the uncertainty of controlling the virus [119]. A meta-analysis regarding the relationship between SARS-CoV-2 and psychological distress (anxiety and depression) conducted by Pappa, Ntella [120] suggested that the combined prevalence of anxiety was 23.2% in 12 studies, while the prevalence of depression was 22.8% in 10 studies. Elbay, Kurtulmus [121] also conducted an online survey to evaluate the psychological responses of healthcare workers, and the results revealed that among all the 442 subjects, 286 showed a depression mood, 224 had anxiety mood, and 182 were under stress during the COVID-19 outbreak. These studies demonstrated a decrease in the number of exercise bouts and the total exercise duration and an increase in psychological distress, including anxiety and depression during the COVID-19 pandemic. Previous studies reported that low frequency of exercise, or even no exercise, and psychological distress are highly associated with immune dysregulation, further causing severe immune-mediated inflammatory diseases and increasing the risk of novel-coronavirus-pneumonia infection [13].

Patients with diabetes often exhibit a high incidence of novel coronavirus pneumonia infection and death, more severe infection symptoms, and a poorer prognosis [37,39]. Some scholars also demonstrated that among the many critical patients with novel coronavirus pneumonia, the percentage of diabetics is higher, which is closely related to the low level of preexisting immunity, lung susceptibility, and inflammation among the diabetic patients [122,123]. Therefore, the lack of exercise and emotional anxiety or depression is of particular concern in the diabetic populations, which are positively correlated with low immunities, high risks of novel-coronavirus-pneumonia infection, more severe infection symptoms, and poorer prognoses. Many scholars studied the effects of non-resistance exercise on immunity and susceptibility to infection, both before and after the COVID-19 pandemic. 

In this study, the related information and challenges faced in high-intensity, moderate-intensity, and low-intensity non-resistance exercises that effectively affected the COVID-19 infection were collated.

### 4.1. High-Intensity Training

Performing regular exercise is beneficial for the prevention of diabetes mellitus and its complications, as well as improving immune response, susceptibility to infection, glycemic control, and insulin sensitivity, ultimately increasing the resistance of patients with diabetes to the COVID-19 virus [124]. However, whether high-intensity exercise, mainly high-intensity interval training, contributes to improving the immunity system of diabetic patients and increasing their resistance to novel-coronavirus-pneumonia infection needs to be discussed.

High-intensity exercise was regarded as an exercise that achieved a targeted heart rate equivalent to >70% of their baseline VO2Max [19]. High-intensity interval training was defined as repeated bouts of high-intensity exercise interspersed with rest periods, which is a very powerful exercise for altering body composition, reducing body weight, and improving cardiovascular conditions, diabetes, and metabolism [125]. High-intensity interval training is a good alternative to moderate-intensity continuous training, and it is also more time-efficient [126]. However, some scholars have suggested that strenuous exercise bouts or intensive training may be detrimental to the normal function of the immune system of the human body, especially cellular and humoral immunity, and may upregulate the level of pro-inflammatory mediators and induce apoptosis or the long-term dysfunction of leukocytes, which is even more severe in diabetic patients [13,127]. However, these claims lack the conclusive support of direct evidence. For example, as stated in the study by Lancaster, Khan [128], prolonged exercise-induced the frequency of leukocytes in blood to increase, but this phenomenon returned to normal after exercise cessation. Therefore, the negative effects of high-intensity exercise were temporary compared to the long-term negative effects of diabetes. On the contrary, according to Sabouri, Hatami [129], high-intensity interval training improved the glycemic parameters and the levels of antioxidant and inflammatory factors (IL-6, C-reactive protein, and TNF-α decreased from around 17.6, 5.4, and 12.7 pg/mL to 13.3, 4.5, and 11.6 pg/mL, respectively). Other scholars have suggested that high-intensity exercise can reduce body weight, adipocytokine production, and the level of C-reactive protein, IL-6, IL-1β, TNF-α, leptin, and resistin [111], increase neutrophil migration to CXCL-8 and the level of anti-inflammatory cytokines such as IL-4, IL-10, and lipocalin [130], improve oxidative stress, improve the ratio of bacteroidetes-to-firmicutes, glycemic control and insulin sensitivity to regulate the immune response, and improve the homeostasis of the internal environment [112,127], thus reducing the risk of infection and diabetes [111]. Denou, Marcinko [112] concluded that high-intensity exercise can prevent the deterioration of inflammation, improve cardiopulmonary function, and maintain the stability of intestinal flora. As indicated in the study of Robinson, Durrer [131], compared to moderate-intensity continuous training, short-term high-intensity interval training demonstrated a better effect in the activation of the anti-inflammatory response, improving glucose control, insulin resistance-induced metabolic disorders, and endothelial function and reducing the expression of toll-like receptors (TLRs) 2 (TLR2) and 4 (TLR4) on lymphocytes and monocytes in patients with type 2 diabetes. Bartlett, Slentz [132] reported that after 10 weeks of high-intensity interval exercise training in diabetic patients, neutrophil dysfunction, glucose control, and insulin sensitivity were improved, while ROS production was significantly reduced, thus decreasing the risk of infections and disease. Huang, Hsu [133] reported that diabetic patients with depression were associated with poor compliance of diabetes management and therapeutic effects, thus aggravating the symptoms of diabetes. However, high-intensity exercise could improve depression and reduce the glycemic levels of diabetics, creating a better general health condition than that of participants without exercise. The study of Simpson, Campbell [13] concluded that the deleterious effects of exercise on immunity were negligible. 

The views of the current study were consistent with those of Sinclair and Abdelhafiz [134], who stated that in the diabetic populations that were potentially infected with COVID-19, maintaining daily exercise can boost immunity, improve glycemic control, and reduce the risk of infection. Thus, it can be concluded that high-intensity exercise can boost immune response, prevent infection, reduce inflammation and improve psychological distress (depression and anxiety), thereby improving the resistance of diabetic patients to COVID-19 infection. However, there are few data on the intervention effect of high-intensity exercise toward diabetic patients who are potentially infected with COVID-19. Therefore, more research is needed to explore the direct effects of high-intensity exercise on COVID-19 infection in patients with diabetes, and in-depth studies regarding the underlying mechanism of this process have also attracted the interest of many scholars.

### 4.2. Moderate-Intensity Exercise

Moderate-intensity exercise is regarded as an exercise that achieves a targeted heart rate equivalent to 40–70% of the individual’s baseline VO2Max [19]. Moderate-intensity exercise is the most commonly performed exercise [135]. However, it is unclear whether higher-intensity exercise is more effective than moderate-intensity exercise in reducing the risk of diabetes, inflammation, and mental disorders, improving the immune system, and preventing COVID-19 infection. There are many controversial arguments and more research is needed to compare the respective advantages of these two types of exercise. Generally, moderate-intensity exercise can be pursued to avoid over-training and overexertion, while enhancing the immune response and reducing the risk of numerous diseases [136], while high-intensity exercise is positively related to increased pro-inflammatory factors and ROS accumulation [137,138], and reduced levels of ainterferon gamma production [139]. Moreover, moderate-intensity exercise is one of the main interventions for people with diabetes and obesity, in addition to providing for appropriate dietary control [140]. However, some studies have argued that moderate-intensity exercise has some disadvantages, such as high levels of apoptosis, poor glycemic control [141,142], and low rates of exercise adherence [143]. Abraha, Chaves [144] stated that the benefits of aerobic exercise seemed to be intensity-dependent, as several studies demonstrated that providing patients/populations with high-intensity aerobic exercise programs was superior to providing low- or moderate-intensity exercise.

However, other scholars have argued that moderate-intensity exercise could provide immuno-protective effects, such as increasing the quantity of natural killer cells, decreasing the concentrations of the tumor necrosis factor, and suppressing the level of inflammation, thus exhibiting its important role in preventing COVID-19 infection and improving diabetes [109]. Barrett, Hayney [145] and Dixit [109] reported that moderate-intensity exercise can effectively prevent the incidence of acute respiratory disease and novel-coronavirus-pneumonia infection. These studies further revealed that moderate-intensity exercise exhibited an active immune function and multiple health benefits, which can be reflected by the increased recirculation of immunoglobulins and higher levels of anti-inflammatory cytokines, neutrophils, NK cells, and cytotoxic T cells [109]. Second, moderate-intensity exercise can positively affect the immune function of the human body, thus further improving its resistance to novel coronavirus pneumonia infection. Chamorro-Vina, Valentin [146] stated that aerobic exercise was associated with increasing levels of NK cells in human circulation, thus enhancing the immune function and inhibiting susceptibility to infection. Khammassi, Ouerghi [147] indicated that moderate-intensity exercise resulted in higher levels of leukocytes, lymphocytes, neutrophils, and monocytes compared to high-intensity exercise, demonstrating better intervention effects in the immune function. Third, moderate-intensity training is beneficial in reducing the emotional burden of patients, such as depression and anxiety mood in COVID-19 infected diabetic patients. As indicated in the study by Borrega-Mouquinho, Sanchez-Gomez [113], both moderate-intensity and high-intensity exercise contributed to the reduction in anxiety, stress, and depression of adults, although better effects seemed to be obtained in the high-intensity exercise groups. Finally, moderate-intensity training reduces pulmonary susceptibility, enhances pulmonary function, and alleviates diabetes-related pulmonary complications in patients with diabetes, thereby improving their resistance to COVID-19 invasion. Halle, Bloch [110] reported that moderate-intensity training can effectively alleviate the pulmonary impairment of athletes caused by novel-coronavirus-pneumonia infection. Both high-intensity and moderate-intensity exercise could suppress excessive inflammation in the respiratory tract, thereby improving immune responses, promoting the regulation of the immune system, and facilitating metabolic health. Therefore, both moderate-intensity exercise and high-intensity exercise have a variety of positive effects on the body, but different types of exercise are generally associated with different positive/negative effects, which, in turn, have different impacts on the ability of individuals to fight against the virus.

### 4.3. Low-Intensity Exercise

Some scholars hold that excessive long-term high-intensity exercise may lead to a downregulation of the immune function. Therefore, low-intensity exercise (<40% VO2Max) is suitable for those patients with chronic diseases and for newcomers to exercise training, especially those with diabetes [19]. Hekmatikar, Shamsi [148] and Piquet, Luczak [149] recommended moderate-intensity training, due to its effectiveness in increasing endorphins and decreasing stress, whereas low-intensity training was more suitable for people with COVID-19. A case study by Hekmatikar, Shamsi [148] demonstrated that low-intensity exercise accelerated the recovery from novel-coronavirus-pneumonia infection. Jesus, Vanhee [150] revealed that heavy exercise was highly related to immune dysfunction, an increased levels of cytokines, and a higher risk of upper-respiratory-tract infections. Moreover, the activity of natural killer cells, T cells, and B cells was greatly reduced. In terms of low-intensity exercises, Jesus, Vanhee [150] reported that long-term adapted exercise was beneficial for the improvement of diabetes mellitus and the illness severity, viral load, and anti-inflammatory effects of patients were significantly enhanced after a period of extended exercise. Similarly, novel-coronavirus-pneumonia-induced inflammation and viral-respiratory infection were also suppressed in these patients. Low-intensity training is also effective in modulating immune responses and suppressing mental disorders, such as depression and anxiety, in normal individuals or diabetic patients, thus contributing to the prevention of and recovery from COVID-19 infection. Rykova, Antropova [151] reported that after 8 weeks of low-intensity exercise, immunocompetent cells, such as CD3+, CD19+, and /CD56+ cells, were activated, while the degree of lymphocyte apoptosis was suppressed. Kimura [152] found that low-intensity exercise played a role in managing blood glucose levels and alleviating the progression of diabetes. As a result, the immune function and resistance to COVID-19 might be increased in diabetes. Moreover, a study by Ji, Yang [153] indicated that low-intensity exercise can alleviate depressive symptoms, while low-intensity training was effective in the treatment of raised anxiety. Tunkamnerdthai, Auvichayapat [154] demonstrated that arm-swing exercise, a kind of low-intensity training, improved lung dysfunction in individuals with diabetes mellitus. The above studies indicate that low-intensity exercises contribute to reductions in anxiety and depression, the prevention of lung damage, and the enhancement of immune response, but the direct link between low-intensity exercises and diabetic patients infected with COVID-19 needs to be further explored.

### 4.4. Exercise Recommendations for Patients Infected with COVID-19

According to the studies of Marcal, Fernandes [155], Abdelbasset, Tantawy [156], and Polero, Rebollo-Seco [157], the performance of at least 2.5 h (0.5 h, 5 days/week) of moderate-intensity exercise per week is recommended, including jogging, brisk walking, rowing, climbing, bicycling, swimming, and skating, or 1.25 h (25 min, 3 days/week) of high-intensity exercise consisting of running, fast uphill walking, climbing, fast swimming, basketball, soccer, volleyball, and jumping rope. Flexibility and balance training (e.g., yoga or tai chi) or other low-intensity exercise (2–3 days/week), such as walking and slow cycling are recommended for elderly diabetic patients in order to promote their health status, achieving excellent glycemic control, enhancing their cardiorespiratory fitness, and improving their psychological status. For high-risk individuals, the use of public places, gyms, sports clubs, and other venues for physical activity is not recommended, and home exercise will be an important way to control blood glucose levels and to ward off SARS-CoV-2 infections.

## 5. Limitations and Future Trends

Diabetic patients, due to their weaker immune protection, higher inflammatory factor levels, more fragile lungs, and poorer psychological conditions, are more susceptible to SARS-CoV-2 infections, further worsening their physical health status. The use of exercise at different intensities may improve the health conditions of these patients and boost the capacity of the human body to resist the SARS-CoV-2 virus. The main shortcoming of this study is the lack of sufficient data to support firm conclusions, which weakens the credibility of the research findings. In addition, most of the previous studies on the effect of exercise intensity on diabetic patients infected with COVID-19 were based on indirect evidence, and there is a lack of direct evidence to deepen the reliability of this study. Finally, only a few of the studies included in this review had large sample sizes, and those studies lacked uniform samples, common clinical conditions and common, methods and evaluations. Thus, there is a need for a better clinical trial with a larger sample size to confirm our findings. Additionally, based on the previously published papers, future research should focus on comparative studies of the interventional effects of different-intensity exercises on diabetic patients infected with COVID-19 to further clarify the optimal exercises for special groups. In addition, the therapeutic value of exercise for diabetic patients infected with COVID-19 should be comprehensively explored from multiple perspectives, such as improvements of immune systems, lung health conditions, and psychological status, as well as the suppression of inflammatory factor levels, which will provide these high-risk individuals with special exercise strategies for different purposes.

## 6. Conclusions

The novel coronavirus pneumonia pandemic has had a negative impact on human health, especially in the diabetic population. The effects of diabetes on individuals with COVID-19 are reflected in the disruption of the immune system, disturbed inflammation levels and blood glucose levels, elevated lung susceptibility and lung damage, and depression and anxiety. Exercise at different intensity levels, including high-intensity, moderate-intensity, and low-intensity exercises, can compensate for the injury caused by COVID-19 in the type 2 diabetic population and have a positive effect on preventing infection and improving prognoses of COVID-19. Low-intensity exercise is recommended for elderly diabetic patients, as it can accelerate their recovery from novel coronavirus pneumonia infection. We suggest that diabetic patients who wish to prevent COVID-19 infection engage in moderate-intensity or high-intensity exercises that have better intervention effects on immune functions. Nevertheless, more in-depth research is needed to assess which exercises are most effective for patients with both SARS-CoV-2 and type 2 diabetes and to elucidate the underlying mechanism of exercise for the treatment of these patients.

## Figures and Tables

**Table 1:** Physical changes induced by diabetes that affect COVID-19 infection.

Model	Age	Research Findings	Reference
HWD	NDM: 62.1 T2DP: 63.4	Neutrophil, LS, IgE, IFN-γ, TNF-α, IL-6 ↑ T, Tc, Th, and NK cells ↓	[51]
Diabetic patients		SMARCD3, PARL, GLIPR1, STAT2, PMAIP1, GP1BA, and TOX genes and PI3K-Akt, focal adhesion, Foxo, phagosome, adrenergic, osteoclast differentiation, platelet activation, insulin, cytokine- cytokine interaction, apoptosis, ECM, JAK-STAT, and oxytocin signaling were the linkage between COVID-19 and Type-2 diabetes	[52]
Diabetic patients	Median age: 55	Creatinine, neutrophil, creatinine, alanine aminotransferase ↑	[53]
HWD	Range from 18 to 60	CD4+ T cells, CD4+/TNF-α, CD4+/IL-2, CD4+/IFN-γ ↓	[54]
HWD	Median age: 47	CD3+, CD4+, CD4+/CD8+ ↑ hs-CRP, LDH, IL-6, CD8+ ↓	[55]
HWD		MMP-7, MMP-9, PDGF and TGF-β ↑ LBP↓	[56]
PUCD	Median age: 61.45	Uncontrolled inflammatory responses, leukocyte, neutrophil-lymphocyte ratio, IL-6, FDP, D-dimer ↑ Pulmonary invasion	[57]
Patients with severe COVID-19	Median age: 64	Susceptible to receiving mechanical ventilation and admission to ICU Mortality, leukocyte, neutrophil, high-sensitivity C reaction protein, procalcitonin, ferritin, IL-2 receptor, IL-6, IL-8, TNF-α, D-dimer, fibrinogen, lactic dehydrogenase, and N-terminal pro-brain natriuretic peptide ↑	[58]
HWD		Depression and anxiety ↑	[59]
HWD	Median age: 40.31	Emotional dysregulation Depression ↑	[60]

HWD: human with or without diabetes; PUCD: patients with uncontrolled and controlled diabetes; MERS-CoV: Middle East respiratory syndrome coronavirus; Tc: cytotoxic T; Th: helper T; NKT: natural killer cells; LS: lymphocyte subpopulations; NDM: non-diabetic participants; T2DP: participants with type 2 diabetes; MMP: matrix metalloproteinase; LBP: lipopolysaccharide-binding protein; TNF-α: tumor necrosis factor; LDH: lactate dehydrogenase.
